# Effects of an informatized system based on the WHO “Safe Childbirth Checklist” combined with oxytocin on post-cesarean section hemorrhage

**DOI:** 10.1097/MD.0000000000049031

**Published:** 2026-06-05

**Authors:** Jia Yang, Chaoyong Wu

**Affiliations:** a Obstetrics VIP Department, Maternity and Child Healthcare Hospital, Keqiao District, Shaoxing, Zhejiang Province, China; b Obstetrics Department, Maternity and Child Healthcare Hospital, Keqiao District, Shaoxing, Zhejiang Province, China.

**Keywords:** cesarean section, informatized management, oxytocin, pelvic floor function, postpartum hemorrhage, WHO Safe Childbirth Checklist

## Abstract

This study evaluates the effectiveness of an informatized system based on the World Health Organization “Safe Childbirth Checklist” combined with standardized oxytocin administration in preventing postpartum hemorrhage (PPH) after cesarean section. A retrospective study included 100 women undergoing cesarean section between January 2023 and August 2025. Patients were divided into a control group (routine care) and an intervention group (informatized checklist plus optimized oxytocin strategy), with 50 cases each. Outcomes included blood loss at 2 hours and 24 hours, PPH incidence, hemoglobin reduction, additional uterotonic use, hospital stay, pelvic floor function, process quality indicators, and quality of life. Multivariate logistic regression was used to identify factors associated with PPH. Compared with the control group, the intervention group had significantly lower blood loss at 2 hours (312.48 ± 70.21 vs 420.56 ± 85.34 mL) and 24 hours (415.76 ± 95.63 vs 540.12 ± 110.45 mL), and a reduced PPH incidence (12.0% vs 30.0%, all *P* < .05). Hemoglobin reduction, additional uterotonic use, and hospital stay were also significantly decreased (all *P* < .05). Pelvic floor function improved, with higher vaginal dynamic pressure and muscle tone scores (*P* < .05). Process indicators, including checklist completion, timely oxytocin administration, and blood loss measurement accuracy, were significantly improved, along with shorter emergency response time and higher staff satisfaction (all *P* < .05). Quality-of-life scores were also higher in all dimensions (*P* < .05). The intervention was identified as an independent protective factor for PPH (odd ratio = 0.157, *P* < .05). The informatized World Health Organization checklist combined with standardized oxytocin use was associated with reduced post-cesarean hemorrhage, improved process quality, and better maternal outcomes. Given the retrospective single-center design and limited sample size, these findings should be interpreted as associative and require confirmation in prospective multicenter studies.

## 1. Introduction

Post-cesarean hemorrhage (PPH) is typically caused by multiple factors, including uterine atony, placental abnormalities, birth canal trauma, and coagulation disorders, among which uterine atony is the most common.^[1,2]^ Therefore, optimizing perioperative management for cesarean section and strengthening strategies for hemorrhage prevention and management are key measures to improve maternal–infant safety and reduce adverse events. Oxytocin, as a first-line drug for the prevention and treatment of postpartum hemorrhage, can effectively promote uterine smooth muscle contraction and has been widely recommended in international guidelines.^[3]^ However, in clinical practice, inconsistencies in dosing and administration timing, along with variability in the skill levels of healthcare providers, lead to significant differences in its preventive effect across institutions. In addition, inadequate standardization of perioperative process management, insufficient communication among healthcare teams, or delays in emergency response may further diminishthe overall effectiveness of oxytocin in preventing post-cesarean hemorrhage.^[4,5]^ Thus, relying solely on pharmacological intervention can no longer meet the current high-quality obstetric management requirements for safety and standardization. To enhance global obstetric safety, the World Health Organization (WHO) issued the “Safe Childbirth Checklist” in 2015, aiming to standardize delivery procedures through structured reminders and promote the implementation of key safety measures. Its principles align closely with modern medical quality control and patient safety systems.^[6]^ Previous studies have demonstrated that the “Safe Childbirth Checklist” contributes to reducing PPH incidence, and oxytocin, as a classical pharmacological intervention, has also been widely recognized for its core role in PPH.^[7,8]^ However, research integrating informatization technologies into the Safe Childbirth Checklist system and combining them with standardized drug strategies such as oxytocin to achieve synergistic benefits in enhancing hemorrhage prevention in the high-risk scenario of cesarean section remains relatively limited. Based on this background, the present study conducted a retrospective analysis with the aim of providing a promotable and replicable comprehensive management model for obstetric clinical practice.

## 2. Materials and methods

### 2.1. General information

This study was approved by the Ethics Committee of Maternity and Child Healthcare Hospital, Keqiao District. A retrospective cohort study was conducted, consecutively enrolling full-term singleton pregnant women who underwent cesarean section in our hospital from January 2023 to August 2025 (KQFY-2025-OBGYN-0826). The control group was enrolled from January 2023 to June 2024, and the intervention group from July 2024 to August 2025. The 2 groups were therefore derived from sequential, nonoverlapping time periods. According to whether an informatized system based on the WHO “Safe Childbirth Checklist” combined with a standardized oxytocin regimen was applied, the participants were divided into an intervention group and a control group, with 50 cases in each group. There were no statistically significant differences between the 2 groups in baseline characteristics, including age, gestational age, parity, body mass index (BMI), indications for cesarean section, pregnancy complications (diabetes, hypertension), and preoperative hemoglobin levels (all *P* > .05), indicating good comparability between the groups (see Table 1). The study protocol was reviewed and approved by the Ethics Committee of our hospital. As this was a retrospective analysis, informed consent was waived.

**Table 1:** Comparison of clinical characteristics between the 2 groups.

Variable	Control group (n = 50)	Intervention group (n = 50)	*Z*/*t*/*χ*^2^	*P*
Age (yr)	29.84 ± 3.12	30.06 ± 3.25	0.345	.731
Gestational age (wk)	38.62 ± 1.21	38.78 ± 1.18	0.669	.505
Primipara	28 (56.00)	27 (54.00)	0.04	.841
BMI (kg/m^2^)	24.36 ± 2.15	24.12 ± 2.23	0.548	.585
Indications for cesarean section			0.218	.975
Social factors	15 (30.00)	14 (28.00)		
Fetal distress	20 (40.00)	21 (42.00)		
Cephalopelvic disproportion	10 (20.00)	11 (22.00)		
Others	5 (10.00)	4 (8.00)		
Gestational diabetes	6 (12.00)	5 (10.00)	0.102	.749
Gestational hypertension	4 (8.00)	3 (6.00)	0.154	.695
Preoperative Hb level (g/L)	115.42 ± 10.12	116.28 ± 9.84	0.431	.668

BMI = body mass index, Hb = haemoglobin.

Inclusion criteria:

① Full-term pregnancy (gestational age ≥ 37 weeks);
② Singleton pregnancy with cephalic presentation;
③ Elective or emergency lower uterine segment cesarean section;
④ American Society of Anesthesiologists physical status classification I–II;
⑤ Complete clinical data and perioperative records available.

Exclusion criteria:

① Severe coagulation disorders or hematologic diseases;
② Obstetric complications likely to cause severe postpartum hemorrhage, such as pernicious placenta previa or placental abruption;
③ Severe dysfunction of major organs, including the heart, liver, or kidneys;
④ Presence of immune system diseases or long-term glucocorticoid use;
⑤ Mental disorders or communication barriers preventing cooperation with the study.

### 2.2. Research methods

The control group received routine postoperative care and treatment after cesarean section. Following fetal delivery, the surgeon administered 20 U of oxytocin via intramyometrial injection based on clinical experience and routine practice, with intravenous infusion added as needed. After delivery, the responsible nurse regularly monitored maternal vital signs, uterine contractions, and vaginal bleeding. All nursing procedures and management strategies followed the existing standard operating procedures of the obstetrics department, without the systematic application of structured checklist tools for process quality control.

In the intervention group, an informatized management system based on the WHO “Safe Childbirth Checklist” was introduced on the basis of routine perioperative management, providing standardized interventions throughout the entire process from admission, predelivery, intrapartum, and 1 hour postpartum to discharge. The system integrated the 29 items of the WHO checklist into a localized format. Through an electronic interface, it automatically generated prompts at different time points, enabling mandatory verification of key steps, automatic documentation, and real-time risk alerts, thereby establishing a “whole-process, traceable, and intervenable” perinatal quality management model. The specific interventions were as follows:

#### 2.2.1. Informatized verification and risk assessment at admission

Upon admission, the informatized system automatically activated the “Admission Verification Module.” The responsible nurse sequentially performed the initial assessment, including vital signs, gestational age, obstetric history, pregnancy complications, and laboratory findings, with each item confirmed and recorded. Based on factors such as anemia severity, placental conditions, uterine contractility, and pregnancy comorbidities, the system automatically generated a hemorrhage risk classification and provided corresponding equipment preparation and management plan reminders tailored to each risk level, ensuring that high-risk women received adequate attention prior to delivery. For any potentially omitted items, the system displayed red alert markers and prevented progression to the next workflow step until completion, ensuring completeness and standardization of key assessment information.

#### 2.2.2. Strengthened process management at key predelivery checkpoints

When the parturient entered the first stage of labor, the system automatically switched to the “Predelivery Verification Module.” Midwives conducted standardized checks of delivery materials, emergency equipment, and neonatal resuscitation devices, while reevaluating maternal and fetal high-risk factors. If necessary, the system automatically provided suggestions for enhanced monitoring or intervention. Through the order-linked function, the system also reminded staff of medication use such as antibiotics or magnesium sulfate to minimize the risk of omission. In addition, midwives were required to complete documentation of education provided to the parturient and family members on warning signs, aiming to enhance their ability to identify abnormalities such as bleeding, abdominal pain, or abnormal fetal movement, thereby achieving shared participation and collaborative risk management during the predelivery stage.

#### 2.2.3. Real-time verification of key intrapartum procedures and standardized oxytocin administration

Immediately after fetal delivery, the system generated a mandatory “Oxytocin Use Within 1 Minute” prompt, requiring healthcare providers to initiate intravenous infusion within the specified time frame and to complete electronic documentation. If delayed, the system automatically issued repeated alerts. The dosage, infusion rate adjustment, and indications for oxytocin administration were all required to be recorded according to system standards. Meanwhile, the quantitative blood loss monitoring module integrated data from both the gravimetric and volumetric methods, automatically generating real-time blood loss values. Combined with built-in risk algorithms, the system issued warnings for rapid increases in blood loss within 10 minutes or for cumulative blood loss exceeding threshold levels, prompting the clinical team to initiate the uterine atony management protocol or the corresponding postpartum hemorrhage emergency plan, thereby improving the timeliness and accuracy of intraoperative hemorrhage detection and intervention.

#### 2.2.4. Maternal–infant safety reevaluation and continuous monitoring within 1 hour postpartum

After delivery, the system automatically entered the “Postpartum 1-Hour Verification Module.” Obstetricians and midwives jointly performed a comprehensive assessment of maternal bleeding, uterine contractions, use of antibiotics and magnesium sulfate, mother–infant skin-to-skin contact, and early breastfeeding, with all findings recorded electronically. A timed reminder function was embedded, prompting documentation of vaginal bleeding and uterine involution every 10 minutes to prevent missed monitoring during busy clinical periods. If abnormalities such as persistent uterine atony or increased bleeding were detected, the system automatically pushed corresponding management steps, thereby strengthening early postpartum risk identification and dynamic management.

#### 2.2.5. Safety verification and individualized health guidance before discharge

On the day of discharge, ward nurses were required to complete each item in the “PreDischarge Verification Module,” including breastfeeding guidance, wound assessment, postpartum rehabilitation recommendations, contraception options, and education on danger signs, with the system automatically marking each item upon completion. Based on the maternal clinical data collected during hospitalization, the system automatically generated an individualized health guidance plan and synchronized it to the follow-up interface, enabling mothers to continue receiving rehabilitation instructions after discharge. High-risk women were automatically included in a prioritized follow-up list to ensure continuity and safety of management before and after discharge.

#### 2.2.6. Informatized quality control and staff training

To ensure standardization and consistency in the application of the checklist, unified training on the informatized system was provided to obstetricians, midwives, and nurses during the study period. The training covered checklist procedures, hemorrhage risk management, standardized oxytocin administration, and system operation. The system’s backend collected real-time data on completion status of all checklist items and automatically generated quality indicators such as execution rate, omission rate, and response time. Department managers held weekly quality-feedback meetings to address delayed checks or low execution rates and to supervise corrective measures. The execution quality of the checklist was incorporated into performance evaluation to encourage healthcare staff to actively comply with standardized procedures in daily practice, thereby improving overall process adherence and safety management.

### 2.3. Observation indicators

#### 2.3.1. Primary outcome indicators

The postpartum blood loss at 2 hours and 24 hours was compared between the 2 groups. Blood loss was quantified using a combined “volumetric + gravimetric” method to ensure accuracy and consistency. The incidence of postpartum hemorrhage was also recorded, with PPH defined as a cumulative blood loss ≥1000 mL within 24 hours after fetal delivery.

#### 2.3.2. Secondary outcome indicators

These included the difference in hemoglobin levels before surgery and 24 hours after surgery (i.e., hemoglobin reduction), blood transfusion rate, use rate of additional uterotonic agents, the proportion of patients requiring interventional hemostatic procedures due to postpartum hemorrhage, and postpartum length of hospital stay.

#### 2.3.3. Pelvic floor function indicators

A vaginal manometric device was used to evaluate pelvic floor muscle strength, and vaginal dynamic pressure (cm H_2_O) was recorded as an objective quantitative indicator of pelvic floor muscle function. In addition, pelvic floor muscle tone at 12 weeks postpartum was assessed according to the perineal muscle testing criteria ([method revised by French National Agency for Accreditation and Evaluation in Health] method) revised by the French National Agency for Accreditation and Evaluation in Health.^[9]^ The score is positively correlated with pelvic floor muscle tension, with higher scores indicating better muscle strength.

#### 2.3.4. Process implementation quality and staff satisfaction indicators

The completeness rate of the WHO Safe Childbirth Checklist, the rate of oxytocin administration within 1 minute after fetal delivery, the implementation rate of quantitative blood loss measurement, and the time from abnormal bleeding detection to activation of the emergency plan were evaluated to reflect the standardization of perioperative processes. In addition, a Visual Analogue Scale^[10]^ was used to assess healthcare staff satisfaction with the new workflow. The Visual Analogue Scale consists of a 10-cm line, with “0 = extremely dissatisfied” and “10 = extremely satisfied” at each end, and staff independently rated their satisfaction, with higher scores indicating greater satisfaction.

#### 2.3.5. Quality of life evaluation

The short form-36 health survey^[11]^ was used to assess maternal quality of life, covering 4 core dimensions: physical, psychological, social, and environmental. Each dimension has a maximum score of 100, with higher scores indicating better quality of life.

### 2.4. Statistical methods

All data were statistically analyzed using SPSS 27.0 (IBM Corporation) and R 4.2.0 software (R Foundation for Statistical Computing). Measurement data were first tested for normality using the Kolmogorov–Smirnov test (sample size ≥ 50). Normally distributed measurement data were expressed as (±), and comparisons between groups were performed using the independent-samples *t* test. Non-normally distributed measurement data were presented as median (interquartile range) and compared using the Mann–Whitney *U* test. Categorical data were expressed as number of cases (%), with comparisons between groups conducted using the *χ*^2^ test or Fisher’s exact test. To control for potential confounding factors, clinically relevant variables such as the intervention, parity, gestational hypertension, and gestational diabetes were directly included in a multivariate logistic regression model to identify independent factors influencing postpartum hemorrhage. A *P* value < .05 was considered statistically significant.

As this was a retrospective observational study, no a priori sample size calculation was performed. To evaluate the adequacy of the sample size, a post hoc power analysis was conducted based on the primary outcome (incidence of postpartum hemorrhage, PPH). Using the observed PPH rates in the 2 groups (30.0% in the control group vs 12.0% in the intervention group), a 2-sided chi-square test with an α level of 0.05 yielded an estimated statistical power of approximately 0.78. These results suggest that the current sample size was sufficient to detect a statistically significant difference in the primary outcome.

## 3. Results

### 3.1. Comparison of clinical data between the 2 groups

There were no statistically significant differences between the 2 groups in terms of age, gestational age, parity, BMI, indications for cesarean section, pregnancy complications, or preoperative hemoglobin levels (all *P* > .05), as shown in Table 1.

### 3.2. Comparison of primary outcome indicators between the 2 groups

Both postpartum blood loss at 2 hours and at 24 hours were significantly lower in the intervention group than in the control group (both *P* < .05), and the incidence of postpartum hemorrhage was also significantly reduced (*P* < .05), as shown in Table 2.

**Table 2:** Comparison of primary outcome indicators between the 2 groups.

Group	n	Postpartum blood loss at 2 h (mL)	Postpartum blood loss at 24 h (mL)	Incidence of PPH (%)
Control group	50	420.56 ± 85.34	540.12 ± 110.45	15 (30.00)
Intervention group	50	312.48 ± 70.21	415.76 ± 95.63	6 (12.00)
*t*/*χ*^2^		6.916	6.019	4.883
*P*		<.001	<.001	.027

PPH = postpartum hemorrhage.

### 3.3. Comparison of secondary outcome indicators between the 2 groups

The intervention group showed a smaller decrease in postoperative hemoglobin, and both the use rate of additional uterotonic agents and the postpartum length of hospital stay were significantly lower compared with the control group (*P* < .05), as shown in Table 3.

**Table 3:** Comparison of secondary outcome indicators between the 2 groups.

Group	n	Hb reduction (g/L)	Blood transfusion rate (%)	Additional uterotonic use (%)	Interventional procedure rate (%)	Postpartum hospital stay (d)
Control group	50	18.36 ± 5.12	5 (10.00)	12 (24.00)	2 (8.00)	4.28 ± 0.82
Intervention group	50	12.28 ± 4.36	1 (2.00)	4 (8.00)	0 (0.00)	3.72 ± 0.65
*t*/*χ*^2^/Fisher		6.393	2.837	4.762	0.475	3.784
*P*		<.001	.092	.029	.51	<.001

Hb = haemoglobin.

### 3.4. Comparison of pelvic floor function and vaginal dynamic pressure between the 2 groups

The vaginal dynamic pressure in the intervention group was higher than that in the control group, and pelvic floor muscle tone was significantly higher in the intervention group (*P* < .05), as shown in Table 4.

**Table 4:** Comparison of pelvic floor function and vaginal dynamic pressure between the 2 groups.

Group	n	Vaginal dynamic pressure (cm H_2_O)	Pelvic floor muscle tone (points)
Control group	50	2.89 ± 0.77	1.24 ± 0.35
Intervention group	50	3.75 ± 1.31	1.77 ± 0.42
*t*		4.001	6.855
*P*		<.001	<.001

### 3.5. Comparison of WHO Safe Childbirth Checklist implementation and process satisfaction between the 2 groups

All implementation indicators of the WHO Safe Childbirth Checklist were significantly better in the intervention group than in the control group, and healthcare staff reported significantly higher satisfaction scores with the workflow (all *P* < .05), as shown in Table 5.

**Table 5:** Comparison of WHO Safe Childbirth Checklist implementation and workflow satisfaction between the 2 groups.

Group	n	Completion rate of WHO checklist (%)	Oxytocin administration within < 1 min after fetal delivery (%)	Accuracy rate of quantitative blood loss measurement (%)	Time from bleeding detection to activation of emergency plan (min)	Healthcare staff satisfaction score (points)
Control group	50	68.05 ± 10.25	60.04 ± 12.53	55.01 ± 15.41	6.48 ± 1.32	7.28 ± 1.12
Intervention group	50	95.24 ± 4.88	90.06 ± 6.22	88.06 ± 7.18	3.12 ± 0.85	9.16 ± 0.84
*t*		16.936	15.174	13.747	15.133	9.495
*P*		<.001	<.001	<.001	<.001	<.001

WHO = World Health Organization.

### 3.6. Comparison of quality of life between the 2 groups

The intervention group achieved higher quality-of-life scores in the physical, social, psychological, and environmental dimensions compared with the control group (all *P* < .05), as shown in Table 6.

**Table 6:** Comparison of quality of life between the 2 groups.

Group	n	Physical (points)	Psychological (points)	Social (points)	Environmental (points)
Control group	50	79.47 ± 2.57	81.73 ± 2.01	82.57 ± 2.61	83.81 ± 2.41
Intervention group	50	88.06 ± 2.85	89.26 ± 1.44	92.06 ± 1.34	90.26 ± 1.05
*t*		15.828	21.534	22.872	17.349
*P*		<.001	<.001	<.001	<.001

### 3.7. Multivariate logistic regression analysis of factors influencing postpartum hemorrhage

To further explore the independent risk factors associated with postpartum hemorrhage, the occurrence of PPH was set as the dependent variable, while intervention measures, parity, gestational hypertension, gestational diabetes, BMI, and preoperative hemoglobin level were included as independent variables. These factors were simultaneously incorporated into a multivariate logistic regression model for analysis. After controlling for potential confounders, the results showed that the informatized system based on the WHO Safe Childbirth Checklist combined with the oxytocin regimen, as well as higher preoperative hemoglobin levels, were independent protective factors against postpartum hemorrhage (*P* < .05), as shown in Table 7.

**Table 7:** Multivariate analysis results.

Variable	β	SE	Wald *χ*^2^	*P*	OR (95% CI)
Intervention group (vs control group)	−1.851	0.653	8.035	.0054	0.157 (0.044–0.565)
Primipara (vs multipara)	0.765	0.527	2.107	.147	2.149 (0.765–6.037)
Gestational hypertension (vs none)	1.127	0.785	2.061	.151	3.086 (0.663–14.377)
Gestational diabetes (vs none)	0.633	0.701	0.815	.367	1.883 (0.477–7.441)
BMI (continuous value)	0.085	0.074	1.319	.251	1.089 (0.942–1.259)
Preoperative Hb level	−0.057	0.029	3.863	.049	0.945 (0.892–1.000)

BMI = body mass index, CI = confidence interval, Hb = hemoglobin, OR = odd ratio, SE = standard error.

## 4. Discussion

Post-cesarean hemorrhage is not only one of the most common clinical complications but also a major cause of cesarean-related maternal mortality. Its occurrence is closely associated with pathological conditions such as uterine atony and coagulation abnormalities. Therefore, when postpartum hemorrhage risk arises, the timely implementation of scientific and systematic nursing interventions is essential. Standardized nursing care can help enhance maternal confidence, stabilize emotions, and improve cooperation with treatment and nursing measures, thereby effectively reducing postoperative complications and lowering the overall risk of postpartum hemorrhage. Oxytocin, as a first-line medication for PPH, has had its pharmacological effects and clinical value confirmed by multiple studies and guidelines.^[12]^ However, the present study found that in the control group relying solely on routine procedures, the incidence of PPH still reached 30.00%, and early postoperative blood loss remained high, indicating that the potential efficacy of oxytocin is difficult to fully realize in a clinical environment lacking standardized implementation and process support.

In this study, the informatized system provided mandatory prompts to initiate oxytocin administration within 1 minute after fetal delivery, increasing the timely administration rate from 60.04% in the control group to 90.06% in the intervention group. This ensured that the pharmacological effect of promoting uterine contraction was exerted within the critical time window, significantly reducing early blood loss and decreasing the reliance on additional uterotonic agents. The smaller decline in hemoglobin levels and the downward trend in transfusion and interventional hemostasis further suggest that standardized oxytocin administration plays an important role in improving postpartum hematologic outcomes. The underlying reasons may be attributed to the following^[13-15]^: the implementation of the informatized checklist system embedded the WHO checklist items into the clinical workflow, enabling dynamic management and closed-loop control of key perinatal steps. Moreover, the intervention group showed significantly higher completeness of checklist implementation, better adherence to quantitative blood loss measurement, and shorter time from abnormal bleeding detection to activation of the emergency plan. These findings indicate that informatization effectively reduces the problem of paper-based checklists becoming a mere formality during execution. The shortened response time reflects faster risk recognition and earlier initiation of interventions, which are crucial quality improvement points in situations where intraoperative or postoperative hemorrhage rapidly progresses. Through automated reminders, process locking, and real-time risk alerts, the informatized system transformed guideline requirements into executable clinical actions, thereby enhancing the consistency and traceability of perioperative management.

Furthermore, the informatized management system and standardized oxytocin use demonstrated a clear synergistic effect. The system ensured the appropriate timing and standardized execution of drug administration, allowing oxytocin to exert its pharmacological effects more effectively, while reducing bleeding events that may result from operator variability, poor communication, or insufficient monitoring.^[16,17]^ The significantly lower incidence of PPH in the intervention group (12.00% vs 30.00%) indicates that the integration of process optimization with standardized pharmacologic intervention achieves clinical outcomes superior to either approach alone. This closed-loop mechanism not only enhances the effectiveness of individual interventions but also improves the overall capacity of the clinical team to respond to obstetric emergencies.

Regarding postpartum recovery indicators, the intervention group had a shorter hospital stay and significantly higher quality-of-life scores across physical, psychological, social, and environmental dimensions compared with the control group, suggesting a positive impact of the combined intervention on overall maternal health status. Reduced bleeding and less severe anemia may promote physical recovery, decrease postoperative fatigue and infection risk, and support breastfeeding as well as early maternal–infant bonding. Improvements in quality of life reflect the contribution of medical interventions not only to short-term clinical outcomes but also to maternal subjective health experience, family function recovery, and social role reintegration.^[18]^

The study also showed better pelvic floor recovery in the intervention group, evidenced by higher vaginal dynamic pressure and improved pelvic floor muscle tone scores. Although cesarean section causes less direct pelvic floor trauma compared with vaginal delivery, postoperative bleeding, restricted mobility, and anemia can delay recovery and affect pelvic floor muscle function. The informatized checklist provided early postoperative rehabilitation guidance, posture adjustment, and activity reminders, helping mothers begin structured rehabilitation training sooner.^[19]^ Additionally, improved physiological conditions resulting from effective hemorrhage control created favorable conditions for pelvic floor recovery.^[20]^ These findings indicate that perioperative quality management influences not only the prevention of acute complications but also long-term postpartum functional recovery.

Furthermore, the increased satisfaction of healthcare personnel provides a positive signal for the sustainable implementation of this intervention model. Staff satisfaction scores were significantly higher in the intervention group, suggesting that the informatized workflow effectively reduces cognitive workload, clarifies role responsibilities, and improves team coordination. Higher satisfaction may enhance adherence to workflow protocols, ensuring the long-term implementation of quality improvement measures in clinical practice.

Multivariate regression analysis indicated that the informatized checklist combined with oxytocin remained an independent protective factor for PPH after controlling for multiple confounders, and higher preoperative hemoglobin levels also showed a protective effect. Preoperative hemoglobin reflects maternal nutritional and hematopoietic reserves; higher levels can enhance tolerance to blood loss and reduce the likelihood of reaching the diagnostic threshold for PPH. These statistical findings support both the independent effect of the combined intervention and the importance of optimizing nutrition and treating anemia during pregnancy as key components of safe delivery management.

In summary, this study demonstrates that integrating the informatized WHO “Safe Childbirth Checklist” system with standardized oxytocin administration can effectively reduce the risk of post-cesarean hemorrhage and promote postpartum functional recovery and improvements in quality of life through the synergistic effects of process standardization and pharmacologic timeliness. This model provides a feasible pathway for enhancing perioperative management in obstetrics. It is recommended that prospective studies and economic evaluations be conducted in broader clinical settings to provide evidence-based support for implementation across different levels of healthcare institutions.

This study still has several limitations. First, it adopted a single-center retrospective design with a relatively limited sample size, and the generalizability of the findings requires validation through multicenter, prospective studies. Second, the implementation of the informatized system depends on hospital information infrastructure and staff training levels, and local adaptation may be necessary during promotion in different institutions. Additionally, this study did not fully evaluate cost-effectiveness, and future research should incorporate economic indicators to comprehensively assess the feasibility and sustainability of the intervention. Finally, the follow-up period for pelvic floor function and quality of life was relatively short, and long-term follow-up data are essential for evaluating the durability of the intervention’s effects. As the intervention group was enrolled in a later time period, potential temporal confounding factors should be considered. Improvements in team experience, enhanced training, and increased familiarity with standardized protocols may have contributed to the observed improvements. Although baseline characteristics were comparable, residual confounding cannot be completely excluded.

## Author contributions

**Conceptualization:** Jia Yang, Chaoyong Wu.

**Data curation:** Jia Yang, Chaoyong Wu.

**Formal analysis:** Jia Yang, Chaoyong Wu.

**Funding acquisition:** Jia Yang.

**Investigation:** Jia Yang.

**Writing – original draft:** Jia Yang, Chaoyong Wu.

**Writing – review & editing:** Jia Yang, Chaoyong Wu.
